# Botulinum toxin treatment for bielschowsky acquired commitant esotropia in adults

**DOI:** 10.1186/s12886-022-02612-7

**Published:** 2022-10-04

**Authors:** Likun Ai, Xiaoli Chen, Ruilin Guo, Jing Li, Jinghui Wang, Yi Feng, Yiqin Guo, Jianan Wang

**Affiliations:** 1 Department of Ophthalmology, Beijing TongRen Hospital, Capital Medical University, Chongwenmen District, No.1 Dongjiaominxiang, 100730 Beijing, China; 2 Department of Ophthalmology, Fuxing Hospital, Capital Medical University, 100038 Beijing, China; 3 Department of Ophthalmology, Beijing Friendship Hospital, Capital Medical University, 100050 Beijing, China; 4 Department of Ophthalmology, Beijing ShunYi KongGang Hostital, 101318 Beijing, China

**Keywords:** Acquired comitant esotropia, Botulinum toxin, Risk factors, Diplopia, Bielschowsky

## Abstract

**Background:**

Many researchers have noticed that there is an increasing trend of Bielschowsky acquired comitant esotropia (ACE) in recent years related to excessive near work, but the exact pathogenesis and treatment methods have not been reported yet. Therefore, we aimed to characterize the clinical features of this ACE in adults and to evaluate the efficacy of *botulinum toxin* (BTX) injections in these patients.

**Methods:**

This was a prospective consecutive case series of 47 patients with Bielschowsky ACE. BTX was injected bilaterally into the medial rectus muscle of 45 patients, and twenty-seven of them (27/45) completed 10 months of follow-up after their last injection. Angle of deviation, fusion, stereopsis, subjective assessment of diplopia were documented before and after BTX treatment, and repeated measures data were compared by the Wilcoxon signed-rank test or Analysis of variance. The relationship between BTX dosage and corrected esotropia was explored by the Regression analysis. Meanwhile, possible risk factors for ACE including time spent on near work, refraction error, patients’ personality, glasses wearing habits and duration of symptoms were recorded and analyzed with General Linear Models.

**Results:**

The patients aged 32.32 ± 10.96 (range 15–53) years spent 8.34 ± 2.38 h on near work each day, and most myope habitually removed their glasses at near. Their chief complaint was distance diplopia, with more significant esotropia at distance (around 20 PD) than at near. This series of patients also exhibited perfectionist tendencies. However, most patients achieved orthophoria after BTX treatment, only with a mild residual esotropia (+ 3.96 ± 5.79 PD), which left them asymptomatic most of the time.

**Conclusion:**

This group of ACE patients was characterized by diplopia with more significant esotropia at distance. Besides excessive near-work, habitually removing myopic glasses and perfectionist tendencies may also contribute to this type of ACE. Fortunately, bilateral BTX injection safely and effectively reduced the esotropia with complete resolution of symptoms, especially for those treated at an early stage.

**Supplementary information:**

The online version contains supplementary material available at 10.1186/s12886-022-02612-7.

## Background

Acquired comitant esotropia (ACE) was used to describe late-onset esotropia with diplopia. Burian and Miller [1] first classified ACE into three categories: (1) Swan type: esotropia precipitated by occlusion of one eye or loss of vision in one eye. (2) Franceschetti type: esotropia presenting with a minimal amount of hypermetropia, often related to physical or psychological shock or exhaustion. (3) Bielschowsky type: esotropia related to excessive near work and uncorrected myopia (< 5 *diopters* (D) [2]. Bielschowsky type of ACE was firstly defined as esotropia at distance but maintained fusion at near without abducens nerve palsy, and then redefined to include higher levels of myopia and constant squint at both near and distance fixations [3]. Though the exact mechanism was not clear, myopia and near work were considered to be crucial in the Bielschowsky type of ACE [4, 5].

However, the prevalence of Bielschowsky type of ACE was low as reported by many researchers [6]. Interestingly, in our clinical practice, we observed an increasing frequency of Bielschowsky ACE in adult patients, and the esotropia angle was relatively small. As *botulinum toxin* (BTX) was less invasive than surgery, literally no muscle and conjunctive scar, we chose BTX as the first line for ACE treatment. Dawson et al. [7] reported that unilateral injection of BTX was effective in the treatment of 14 children with ACE, but the authors did not give details on the specific type of ACE. In contrast, the purpose of our study was to describe the characteristics of Bielschowsky ACE in adult patients, and evaluate their responses to bilateral injection of BTX.

## Methods

The protocol was approved by the Ethics Committee of the Eye Center in Tongren Hospital, affiliated with Capital Medical University. A prospective study was performed on a series of consecutive patients with Bielschowsky ACE presenting to the Pediatric Ophthalmology and Strabismus Division between 2015 and 2018. The study was conducted in accordance with the tenets of the Declaration of Helsinki and approved by the Institutional Review Board.

Clinical data.

Inclusion criteria included a new onset of comitant esotropia with diplopia, reported near work of more than 4 h per day, and no history of prior strabismus or systemic diseases. Patients with abducens nerve palsy or neurologic diseases of any kind were excluded, as well as patients with bifocal or multifocal glasses. All patients were referred to the neurology department at the first visit. A total of 47 patients were included. Their name, occupation, age at presentation, sex, race, hours of near work performed each day, whether removing myopic glasses at near, and duration of symptoms (diplopia) as well as the tendency of perfectionism on a scale from 0 to 4 were collected.

Specifically, we asked each participant to rate the severity of diplopia in each visit, also on a scale from 0 to 4; 0: no diplopia neither distance nor near, 1: occasional diplopia only at distance, 2: occasional diplopia both at distance and at near, 3: diplopia both at distance and at near most of the time, 4: constant diplopia at distance and at near.

All patients received a complete eye examination including cycloplegic refraction, ratio of accommodative convergence/accommodation (AC/A) using the gradient method, angle of deviation measured in prism diopters by alternate prism cover test, and fusional amplitudes and stereopsis determined by the synoptophore.

Treatments.

As a first line treatment, all patients were recommended to refrain themselves from near work. For patients whose symptoms were not improved, BTX (BTXA, Hengli, Lanzhou Institute of Biological Products) was injected into the bilateral medial rectus muscles by author LA using electromyography. During the follow-up period, BTX injections were repeated when diplopia recurred and the patient could not tolerate it.

### Statistical analysis

Statistical analysis was performed using SPSS 22 (SPSS, Inc., Chicago, IL, USA). All values were reported as mean ± standard deviation unless otherwise specified. Data pre- and post-injection were compared by the Wilcoxon signed-rank test or Analysis of variance for repeated measures data. General Linear Models and Regression analysis were used to investigate the risk factors and BTX dosage for Bielschowsky ACE, respectively. A level of 0.05 was required to demonstrate statistical significance.

## Results

### Clinical characteristics and risk factors for patients with Bielschowsky type of ACE

Forty-seven patients were included with 24 females and 23 males. The mean age at diagnosis was 32.32 ± 10.96 (range 15–53) years. Diplopia at distance was the chief complaint for all patients, which was often fleeting at first, beginning after a period of prolonged near work, and resolved when patients stopped working and relaxed. However, symptoms increased in severity over time, with both increasing duration and frequency, resulting in constant diplopia at both distance and near in some patients.

As shown in Table 1, although most patients complained of diplopia only at distance, almost all patients presented with esotropia both at distance (+ 22.11 ± 8.55 PD, range, + 8 PD to + 45 PD) and near (+ 13.47 ± 6.62 PD, range, 0 to + 35 PD). The esotropia angle was significantly larger at distance than near (p < 0.001). Overall, the AC/A ratio was slightly lower and the majority of patients passed the stereopsis test. Meanwhile, most patients (37/47) were myopic and wore glasses, but 67.57% (25/37) habitually removed their glasses when performing near work.

**Table 1:** Clinical characteristics of patients with Bielschowsky type of ACE

Variables	N = 47
Sex (male/female)	23/24
Age at diagnosis (years)	32.32 ± 10.96
Duration of diplopia (months)	17.77 ± 20.65
Follow-up period (months)	11.34 ± 7.18
Right refraction (Diopter)	-3.97 ± 2.42
Left refraction (Diopter)	-3.98 ± 2.21
Deviation at distance (PD)	+ 22.11 ± 8.55
Deviation at near (PD)	+ 13.47 ± + 6.62
Diplopia (+)/Diplopia (-)	47/0
Stereopsis (+)/Stereopsis (-)	32/15
Convergence (PD)	8.30 ± 3.75
Divergence (PD)	-6.67 ± 5.18
AC/A ratio	2.48 ± 1.22
Near-work time	8.34 ± 2.38
Patient’s personality score	3.41 ± 0.78

*PD* Prism diopters; *AC/A* Accommodative convergence/accommodation

Due to the occupational requirements, most patients who were programmers, accountants, teachers, lawyers, physicians and general office staff had to read or write during the majority of their working hours. And the time spent on near work was reported to be an average of 8.34 ± 2.38 h(range 4 to 20 h)per day. In the self-evaluation of the personality of perfectionism (score range 0 to 4), the average score in our group was up to 3.41 ± 0.78. Most patients admitted that they took their work very seriously, and three patients (6.4%) were diagnosed with obsessive-compulsive disorder and had to take medication to control their symptoms.

AIn order to further explore the risk factors for ACE, the relationship between the distance deviation of ACE patients and possible risk factors was analyzed, including refractive error, near-work hours per day and duration of diplopia. Pearson’s correlation analysis or Spearman’s correlation analysis was chosen, depending on the data were normally distributed or not. As a result, only duration of diplopia was statistically correlated with the severity of esotropia (R = 0.41, p = 0.01, Pearson)

## Treatment of BTX injection significantly improved orthotropic appearance and diplopia in ACE patients

Among all of the 47 ACE patients, two (2/47) patients’ symptoms were resolved by near-work refrainment. The remaining 45 patients underwent BTX injections bilaterally into the medial rectus muscles, up to three injections. A total of twenty-seven (27/45) patients completed 10 months of the recommended follow-up at the time of this publication, and their characteristics were displayed in Table 2.

**Table 2:** Comparisons between Pre- and Post-injection of BTX in 27 ACE patients who completed 10-month follow up

Patients No.	Age (years)	Gender	Duration of esotropia (months)	Esodeviation (PD) (near)		Esodeviation (PD) (distance)		Total BTX units for bilateral injection	Number of injections in total	Score of diplopia		Presence of stereopsis	
				Pre-BTX/Post-BTX		Pre-BTX/Post-BTX				Pre-BTX/Post-BTX		Pre-BTX/Post-BTX	
1	16	F	3	30	0	40	10	6	1	4	1	-	+
2	27	M	6	15	2	15	3	3	1	4	0	+	+
3	28	F	24	20	0	20	2	3	1	4	0	+	+
4	37	F	24	15	10	25	14	3.5	1	4	0	+	-
5	15	M	6	20	0	20	0	4	1	4	0	+	+
6	34	M	12	10	5	10	8	3	1	3	0	+	+
7	33	M	12	20	0	20	0	3	1	4	1	-	+
8	33	M	24	0	0	10	4	2.5	1	1	0	+	+
9	23	M	14	15	0	20	0	4	1	4	0	-	+
10	34	F	24	15	0	20	0	4	1	4	0	+	+
11	21	F	24	25	10	25	10	7.5	2	4	0	-	-
12	19	F	36	30	15	40	25	13.5	3	4	2	-	-
13	18	M	12	15	4	20	0	4	1	4	1	+	+
14	34	M	36	15	0	20	8	3.5	1	4	0	+	+
15	23	M	8	15	0	25	0	3.5	1	4	0	-	+
16	40	M	11	15	5	13	5	4	1	4	1	+	+
17	53	F	12	20	0	25	3	2.5	1	4	0	+	+
18	30	M	12	15	5	15	5	3.5	1	3	0	+	+
19	48	F	24	15	0	20	0	3.5	1	4	0	+	+
20	51	M	1	10	0	10	0	3	1	3	1	+	+
21	22	M	12	20	5	15	0	2.5	1	4	1	+	+
22	25	M	12	35	0	40	3	4	1	4	2	-	+
23	17	M	6	8	0	15	7	3.5	1	3	1	+	+
24	21	F	60	5	0	8	0	3	1	1	0	+	+
25	27	M	12	15	0	15	0	2.5	1	4	0	+	+
26	30	F	2	35	0	40	0	5	1	4	2	-	+
27	40	F	6	10	0	25	0	3.5	1	4	0	+	+

*PD* Prism diopters; *F* Female; *M* Male; *BTX* botulinum toxin

**Fig. 1:**
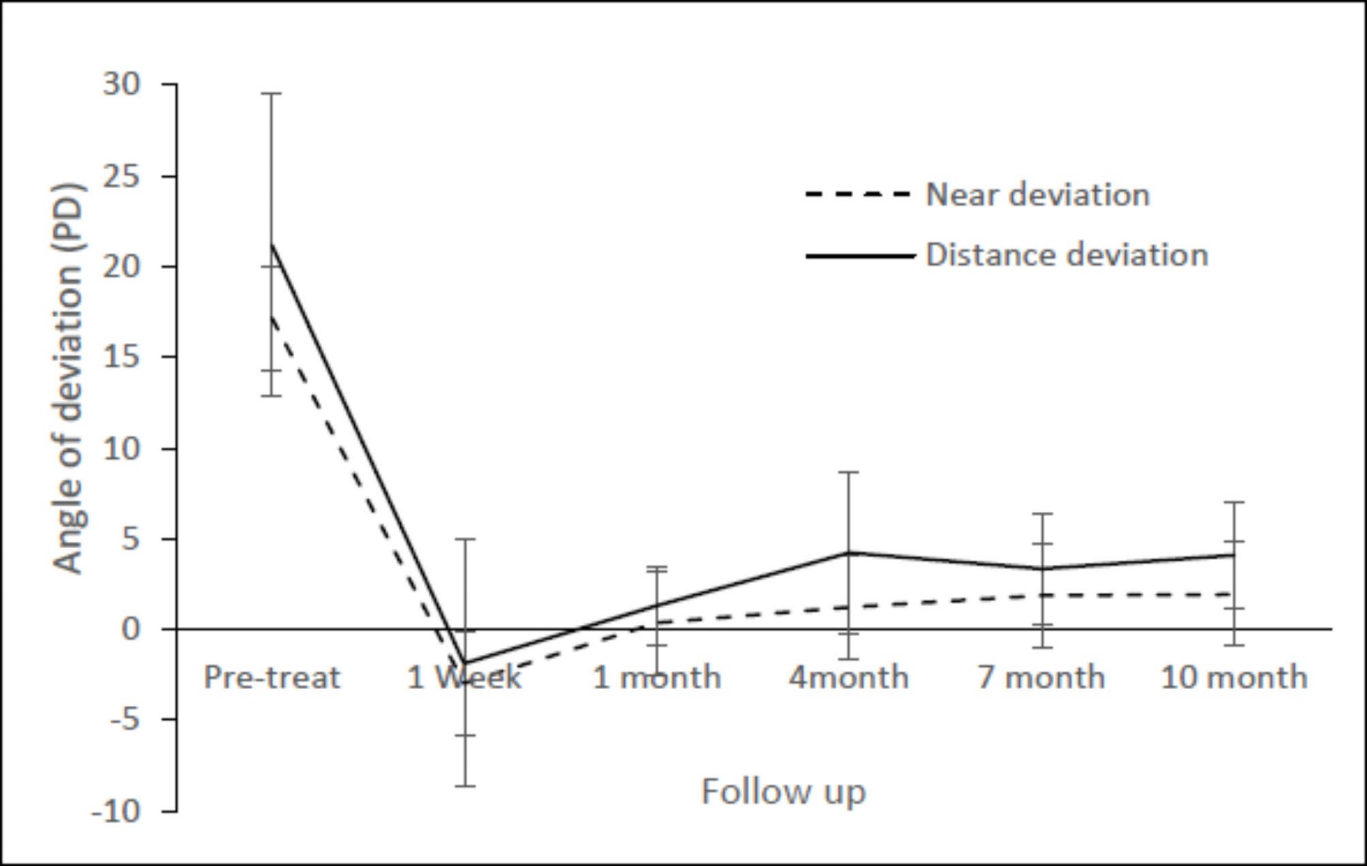
Magnitude of esotropia before and after BTX treatment in the 27 patients who completed 10-month follow up. *PD* prism diopters

Orthotropic appearance was achieved in most cases during the follow-up period. As shown in Fig. 1, the average angle measured 1 week after injection was − 1.89 ± 9.22 PD for distance. Some patients complained of crossed diplopia in the first one to two weeks due to the overcorrection, but almost all of the patients were satisfied with the amelioration of distance diplopia at the one-month follow up. Although some patients showed a mild return of esotropia at the end of the 10-month follow up (Fig. 1), the residual esotropia (for distance, + 4.07 ± 3.86 PD; for near + 1.93 ± 2.87 PD) was significantly smaller than that before treatment (distance + 21.15 ± 9.42 PD, Z=-4.54, p < 0.0001; near + 17.15 ± 8.32 PD, Z=-4.47, p < 0.0001).

For the subjective assessment of diplopia, the average score reduced from 3.63 ± 0.84 before treatment to 0.48 ± 0.70 at the last visit (p < 0.001), and 62.96% (17/27) patients gave the lowest score (0) at the last visit. Some patients (7/27) reported occasional diplopia with prolonged near work, but symptoms resolved when they relaxed. Divergence fusional ranges were sufficient to overcome the residual esotropia in most patients but one(case No. 12). This patient complained of severe diplopia with esotropia (+ 35 PD/+40 PD near/distance) 3–4 months after each injection (a total of 3 BTX injections) and might need surgical correction. Two patients (2/27) came back for recurrent diplopia after the 10-month observation. No serious side effects were noticed, and only 11.11% (3/27) of patients experienced temporary blepharoptosis, which resolved spontaneously in 2–6 weeks.

## Treatment of BTX injection increased binocular stereopsis in ACE patients

The pass rate of stereopsis test increased from 70.37% (19/27) before treatment to 88.89% (24/27) by the end of follow up. Meanwhile, the fusional convergence and divergence ranges did not change significantly over the 10-month follow-up (Table 3).

**Table 3:** Convergence/divergence amplitudes of 27 patients who completed 10-month follow up after the last BTX injection

	Initial	7th month	10th month	Significance
Convergence (PD)	7.61 ± 4.06	8.40 ± 3.93	8.63 ± 2.77	F = 0.39, p = 0.68
Divergence (PD)	6.32 ± 4.88	6.33 ± 2.12	5.60 ± 1.25	F = 0.53, p = 0.60

*PD*, prism diopters

## Dose-response effect for treatment of BTX injection in ACE patients

**Fig. 2:**
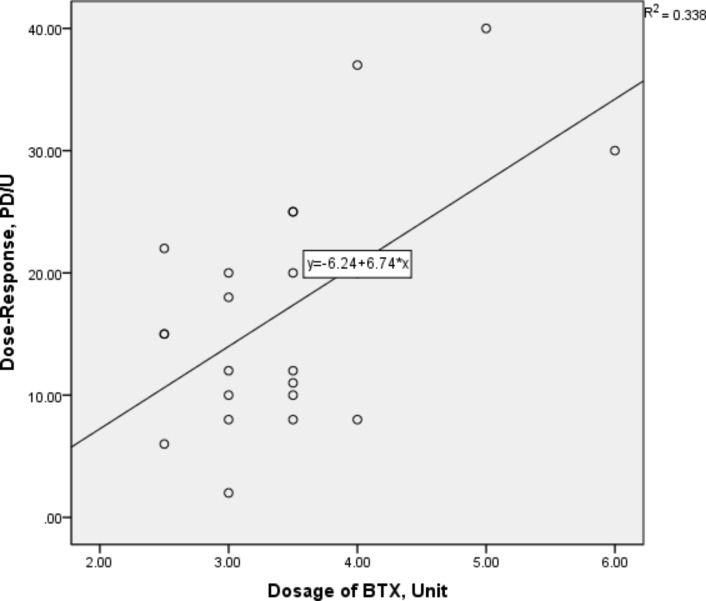
Dose-response model for BTX treatment. The Regression analysis showing the relationship between the angle of corrected esotropia (PD) and the total dosage of bilateral injection of BTX. *PD* prism diopters; *U* unit

The dose-response relationship between BTX treatment and the corrected esotropia was significant(Figure 2 R^2^ = 0.338, p = 0.002) demonstrated by the Regression analysis. The linear regression model was used to predict the eye alignment response to a certain amount of BTX, which suggested that BTX injection was a relatively predictable treatment for this Bielschowsky type of ACE.

## Discussion

ACE does not prevail in esotropia, but it is annoying due to the diplopia, especially in adults [8]. Worldwide, strabismologists have started to notice an increasing trend of ACE in recent years [9]. As one of the largest eye centers in China, we experienced a growing trend of near-work-related ACE (Bielschowsky type). In our study, a total of 47 patients with Bielschowsky ACE were included and their clinical characteristics were analyzed.

As a progressive acquired esotropia, Bielschowsky ACE was characterized by intermittent diplopia at distance, with a small angle esotropia (generally less than 10 PD) at the early stage. Symptoms could be worsened with prolonged near work and vice versa. Gradually, constant diplopia could be observed both at distance and near, mainly a greater esotropia at distance, averaging around + 20 ∆. Meanwhile, almost all patients spent plenty of time on near work (8.34 ± 2.38 h). Some patients were myopia and used to removing their myopic glasses while doing near work. Additionally, most patients self-identified as perfectionists.

It was self-evident that ACE patients in our study were not fully compliant with but quite close to the criteria of Bielschowsky type. Bielschowsky type was believed to be related to myopia and near work [10, 11], whereas some of the patients in our study had emmetropia and even mild hyperopia. In addition, Bielschowsky type has not been reported to have perfectionist personality. Therefore, maybe it is more appropriate and accurate to call them Bielschowsky-like type of ACE. However, the majority of patients in our study were myopic and we were inclined to categorize them as Bielschowsky type for the sake of easy understanding.

Our results displayed that long duration of symptoms (diplopia) was a risk factor for this Bielschowsky type of ACE. Moreover, we believed that myopia and long hours on near work were also risk factors for ACE, though the statistical analysis showed no significance, which could probably due to the relatively small sample size. Previous researches indicated that prolonged near work was likely to reduce amplitude of accommodation and fusional convergence due to fatigue [11, 12]. The decreased accommodation capability might lead myopic patients to remove their glasses to reduce requirement of accommodation when doing near-work tasks, so less accommodative convergence was elicited. Consequently, to hold clear binocular vision at near, other components of convergence including fusional, tonic and proximal convergence needed to be increased which might persistently increase the tonus of the medial rectus muscles [1, 13, 14].

We speculated that those increased non-accommodative convergences kept the eyes converged to achieve single vision at near, but high tonic convergence might not be able to relax even when patients relax their accommodation by looking at distant objects. As a result, diplopia presented at distance in the beginning and eyes were progressively driven into a more esotropic posture, which might reflect the increase of tonic convergence overtime. Interestingly, ACE patients in our study showed a slightly lower AC/A ratio, reflecting a lower accommodative convergence response in this group, which also supported our hypothesis. Furthermore, none of the patients reported psychological shock before the onset of diplopia, but most of them self-identified as perfectionists. Perfectionism might keep the nervous system constantly alert, consequently contributing to the increased tonus of the medial rectus muscle [15, 16], which needs further investigation.

Since high tonus of the medial rectus muscle was supposed, we tried first non-invasive therapy and then BTX injection to reduce the muscle’s tonus level for the treatment of this Bielschowsky type of ACE. Our study suggested that reducing the near-work time might be sufficient to alleviate symptoms in mild cases and BTX was effective for most cases. BTX injection has become more and more popular as a safe and repeatable method for strabismus treatment since first described by Scott [17]. Theoretically, BTX can effectively reduce the tonus of the medial rectus muscles in ACE, helping balance the tonus of medial and lateral recti, consequently improving the symptom of diplopia. Dawson et al. reported that 79% patients (11/14) gained satisfactory ocular alignment with BTX therapy and refrained from squint surgery [7]. In consistence with this, our results of BTX treatment were quite encouraging in the management of Bielschowsky type of ACE patients, and one injection was enough for most patients (25/27) to regain normal binocular vision. Moreover, in our study, due to the small angle of esotropia, we only intended to achieve a slight overcorrection which turned out to be effective for this Bielschowsky ACE. Since the long-term effects of BTXA injection are related to the degree and duration of transient overcorrection, more data and longer follow-up time are required to draw any conclusions about the overcorrection. However, unlike Dawson’s research [7], which suggested that 2.5 units of BTXA injected into unilateral medial rectus muscle was rational for ACE treatment, there was a linear dose-response relationship between BTX and Bielschowsky ACE in our study. The difference may be related to different patient groups, as adults and children were investigated in our study and Dawson’s study, respectively. This warrants further investigation on the optimal dosage of BTX injections for ACE treatment.

Additionally, divergence insufficiency esotropia (DIE) as an important differential diagnosis should be carefully identified. Duane described this acquired esotropia a century ago [18], and DIE matched part of the symptoms in our group of ACE patients. The diplopia was mainly at distance in DIE, also fleeting at first and aggravated in duration and frequency over time. Additionally, the angle of deviation was also modest (12–20 PD), greater at distance than near. However, DIE was less sensitive to BTX treatment and posed weakness in distance fusional divergence amplitude [19]. However, DIE was primarily observed with degeneration of the suspensory ligaments of the lateral rectus in elderly people and now is treated as “age-related distance esotropia” or “sagging eye” by many researchers [20, 21]. Some investigators noticed that DIE occurred predominantly in females and Caucasian patients [22]. Unlike DIE, most patients in our study were Asian, with a fairly equal mix of both males and females, much younger age, and no signs of sagging eye syndrome such as baggy eyelids, superior sulcus deformity or aponeurotic blepharoptosis, making them unlikely to be classified as this age-related DIE.

There are several limitations in our study. First, our sample is relatively small due to the low prevalence of this Bielschowsky ACE, and consequently we could not specifically investigate the impact of different types of near work (including smartphone use, computer using and reading) on ACE. To clarify this, we plan to recruit more ACE patients in the future studies. Then magnetic resonance imaging is not performed to look for pulley heterotopia or other signs of sagging eye syndrome, which is advocated by many researchers [23]. However, all patients in our study were young and possible neurological diseases were ruled out by neurologist. Therefore, our results are reliable and magnetic resonance imaging should be considered in the future studies. Another limitation is that the follow-up period may be not long enough to investigate the long-term effect of BTX injection, which requires further research.

## Conclusion

This Bielschowsky type of ACE appears to be much more popular than before, characterized by excessive near work, habitually without glasses (for myopes) and perfectionism. Accordingly, refraining from near work and wearing glasses all the time (if myopic) might be suggested. Moreover, bilateral BTX injection is a safe and quite effective treatment for this Bielschowsky ACE, especially at an early stage.

## Electronic supplementary material

Below is the link to the electronic supplementary material.

Supplementary Material 1

## Data Availability

The datasets used and/or analyzed during the current study are available from the corresponding author on reasonable request.

## Declarations

### Authors’ information

ALK is a professor and specialist in Strabismus and Pediatric Ophthalmology, working in the department of Ophthalmology, Beijing TongRen Hospital, Capital Medical University, Beijing 100730, China.

## List of abbreviations

ACE: Acquired comitant esotropia.
BTX: Botulinum toxin.
D: diopters.
PD: Prism diopters.
AC/A: Accommodative convergence/accommodation ratio.
DIE: divergence insufficiency esotropia.
