# Supplementary material for: Botulinum toxin treatment for bielschowsky acquired commitant esotropia in adults
Source: BMC Ophthalmol. 2022 Oct 4;22:395. doi: 10.1186/s12886-022-02612-7 (PMC9533611; doi:10.1186/s12886-022-02612-7)
Supplement: Supplementary file 1 — Supplementary Material 1 [file 12886_2022_2612_MOESM1_ESM.docx]

**Supplementary Table 1**

| Patient’s No. | Spherical equivalent refraction of right eye (Diopter) | Spherical equivalent refraction of left eye (Diopter) | Follow-up period (months) |
| --- | --- | --- | --- |
| 1 | -3.75 | -2.625 | 25 |
| 2 | -5.125 | -5 | 25 |
| 3 | -2.75 | -3.5 | 23 |
| 4 | -4.5 | -4.25 | 22 |
| 5 | -2.25 | -2 | 22 |
| 6 | -4.875 | -4 | 22 |
| 7 | 0.375 | -0.25 | 22 |
| 8 | -2.5 | -2.625 | 19 |
| 9 | -6.25 | -5.5 | 19 |
| 10 | -1.875 | -5.25 | 19 |
| 11 | -0.5 | -0.5 | 10 |
| 12 | -9 | -8.25 | 10 |
| 13 | -4 | -5.125 | 19 |
| 14 | -0.75 | -0.5 | 16 |
| 15 | -4.75 | -5 | 16 |
| 16 | -8.75 | -4.575 | 16 |
| 17 | -3.25 | -3.5 | 16 |
| 18 | 0 | 0.25 | 16 |
| 19 | -5.25 | -4.875 | 16 |
| 20 | -4 | -4.25 | 13 |
| 21 | -0.875 | -3.375 | 13 |
| 22 | -4.625 | -4.25 | 13 |
| 23 | -6.375 | -6.25 | 10 |
| 24 | -7.125 | -8 | 10 |
| 25 | -7.25 | -6.125 | 10 |
| 26 | -6.25 | -6.25 | 10 |
| 27 | -3.75 | -3.625 | 10 |
| 28 | -6.625 | -6.375 | 7 |
| 29 | -3.5 | -3.5 | 7 |
| 30 | -4.5 | -3.25 | 7 |
| 31 | -4.25 | -4 | 7 |
| 32 | -2.25 | -1.5 | 7 |
| 33 | -3.35 | -2.95 | 7 |
| 34 | -2 | -2.5 | 7 |
| 35 | 0.75 | -0.25 | 6 |
| 36 | -0.25 | 0 | 4 |
| 37 | -4.25 | -3.6 | 4 |
| 38 | -7 | -7.75 | 4 |
| 39 | -0.25 | -0.75 | 4 |
| 40 | -3.375 | -3.75 | 4 |
| 41 | -5.75 | -6.25 | 4 |
| 42 | -7.75 | -7.75 | 4 |
| 43 | -3 | -2.75 | 4 |
| 44 | -5.375 | -6.5 | 1 |
| 45 | -6.25 | -5.75 | 1 |
| 46 | -4 | -4 | 1 |
| 47 | -3.5 | -4.5 | 1 |
